# Postoperative Pyoderma Gangrenosum Following Carpal Tunnel Surgery: A Case Report and Review of the Literature

**DOI:** 10.7759/cureus.54590

**Published:** 2024-02-20

**Authors:** Yusuf Can Edek, Muhammed Kaan Temirkaynak, Berkay Temel, Melike Urgancı, Betül Öğüt, Esra Adışen

**Affiliations:** 1 Dermatology, Gazi University, Ankara, TUR; 2 Dermatology, Ankara Training and Research Hospital, Ministry of Health, Ankara, TUR; 3 Pathology, Gazi University, Ankara, TUR

**Keywords:** postoperative pyoderma gangrenosum, diagnosis of pyoderma gangrenosum, carpal tunnel syndome, carpal tunnel surgery, pyoderma gangenosum

## Abstract

Pyoderma gangrenosum (PG) is a neutrophilic dermatosis characterized by painful ulcerated lesions. Postoperative PG, which typically begins with erythema and severe pain within two weeks after surgery, progresses into ulcerated lesions. It is often misdiagnosed as it resembles necrotizing skin infections, resulting in delayed treatment. Cases of postoperative PG located in the upper extremity are uncommon. In this case report, we discuss a male patient who developed postoperative PG after carpal tunnel surgery.

## Introduction

Pyoderma gangrenosum (PG) is a noninfectious neutrophilic dermatosis characterized by painful ulcerated lesions with violaceous borders. The diagnosis of the disease can be made based on typical clinical findings and medical history, once other potential conditions have been ruled out. Although histopathological examination of PG lesions is nonspecific, histopathological evaluation in patients is important to exclude other etiological factors. In the histopathological examination of the lesions, ulceration, edema in the epidermis, intense neutrophilic infiltration in the epidermis and dermis, dermal abscess formations, dermal necrosis, thrombosis, and hemorrhage in the vascular structures can be observed. The frequent association of PG with systemic comorbidities such as inflammatory bowel diseases, arthritis, and lymphoproliferative diseases may require detailed questioning of patients regarding these comorbidities and a multidisciplinary approach to disease management. In the management of the disease, local treatments (e.g., wound care and negative pressure therapy, topical steroids, calcineurin inhibitors) and systemic treatment agents (e.g., systemic steroids, cyclosporine, azathioprine, methotrexate, mycophenolate mofetil, dapsone, and biological agents can be used). While ulcerative PG is the most common and characteristic form of PG, postoperative PG cases have recently attracted increasing attention in the literature [1-4].

Postoperative PG is a type of PG that usually begins with erythema and severe pain in the operation area within two weeks after surgery and develops into an ulcerated lesion over time. It may occur as a surgical complication and because of the similarity of the clinical appearance with necrotizing skin infections, patients are often misdiagnosed, and treatments may be delayed [5,6].

In this case report, we present a male patient who was evaluated with a rapid-onset ulcerated lesion on the wrist following carpal tunnel surgery and had a delayed PG diagnosis.

## Case presentation

**Figure 1:**
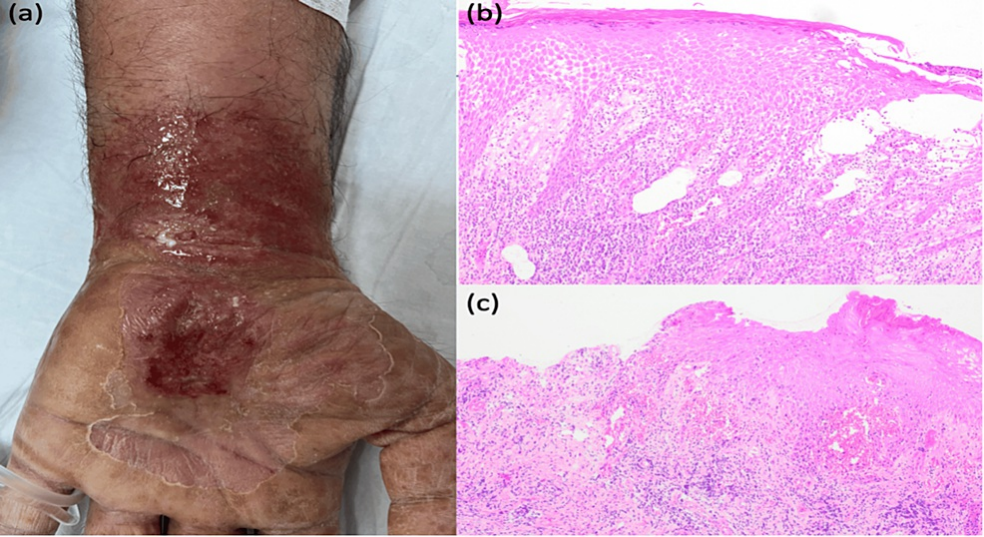
(a) Draining ulcerated lesion measuring 6x4 cm in size at the wrist level on the left arm, (b,c) histopathological evaluation; focal ulceration in the epidermis, acanthosis, spongiosis, neutrophil-rich inflammation in the intact epidermis, lichenoid-style mixed type inflammation in the dermis (H&E, x100, x100).

A 42-year-old male patient presented to our outpatient clinic with a painful, draining lesion on his left wrist. He explained that he had undergone surgery on his left wrist six months ago for carpal tunnel syndrome, and a week after the surgery, a small painful papule appeared in the surgical area, which gradually developed into an ulcerated lesion. Despite receiving topical and systemic antibiotics, the patient's lesion continued to grow and discharge. The patient's medical history was significant for rheumatoid arthritis (RA) treated with leflunomide and diabetes mellitus (DM) treated with metformin. A dermatological examination revealed a 6x4 cm ulcerated lesion on the left arm at the wrist level. Histopathological evaluation of a 4 mm punch biopsy taken from the lesion showed focal ulceration, acanthosis, spongiosis, neutrophilic infiltration in the epidermis, and lichenoid-style mixed-type inflammation in the dermis (Figure 1). The patient met one major and five minor criteria of the Delphi diagnostic criteria, resulting in a Paracelsus score of 17. Surgery was the only identified triggering factor. He was diagnosed with postoperative PG, and systemic steroid (1 mg/kg/day) treatment was started in the follow-up. The patient's complaints regressed without any recurrence or side effects, and steroid treatment was tapered.

## Discussion

Postoperative PG often occurs following breast and abdomen surgeries. Although the pathogenesis of PG is not fully understood, it is thought that interleukin 8 (IL-8), which increases with surgical trauma and pathergy response and stimulates neutrophil activation, may be effective in the postoperative PG pathogenesis. Increased T-helper 17 cells and IL-23 levels and reduced regulatory T-cell levels can play a role in the pathogenesis. Additionally, genetic mutations in the genes encoding methylenetetrahydrofolate reductase and janus kinase 2 enzymes have been considered predisposing factors [7-9]. Postoperative PG cases have been found to have fewer accompanying systemic comorbidities compared to patients with ulcerative PG. While the frequency of accompanying systemic comorbidity in ulcerative PG was found to be 50-78% in various studies, Tolkachjov et al. found the frequency of comorbidity in the postoperative PG group to be 22% in their 20-year retrospective analysis [6]. Our patient had RA, one of the PG-related comorbidities. In postoperative PG, immunosuppressive agents are at the forefront of the treatment, and the regression of the lesions with immunosuppressive treatment is one of the characteristic features of PG. The therapeutic options for ulcerative PG and postoperative PG are similar. The first step in treatment involves the use of systemic corticosteroids and cyclosporine. Additionally, other options such as azathioprine, methotrexate, mycophenolate mofetil, dapsone, and biological agents can be considered for treatment [5,6].

Lesions located in the upper extremity have been rarely reported. In their study, Tolkachjov et al. highlighted that only one out of 18 postoperative PG patients had an upper extremity-located lesion [6]. There are a few postoperative PG cases after carpal tunnel surgery in the literature (Table 1) [10-13]. Mehdiyev et al. reported a postoperative PG developing after carpal tunnel surgery in a female patient with a known PG history [10]. Ruebhausen et al. published a postoperative PG case following carpal tunnel surgery, systemic steroids, dapsone, vacuum-assisted closure (VAC), and skin grafts, and, upon no treatment response, amputation was performed [12].

**Table 1:** Postoperative pyoderma gangrenosum cases developed after carpal tunnel surgery reported in the literature. F: female, M: male, HPL: hyperlipidemia, PG: pyoderma gangrenosum, DM: diabetes mellitus, RA: rheumatoid arthritis, VAC: vacuum-assisted closure, NA: not available

Case	Age/sex	Comorbidities	Interval between surgery and disease (day)	Antibiotherapy history	Debridement history	Wound culture	Treatment
Mehdiyev et al. [10]	81/F	HPL, PG, goiter, hyperuricemia, urinary incontinence	7	+	+	Sterile	Systemic steroid, antibiotic
Geerards et al. [11]	60/M	NA	NA	+	+	Sterile	Systemic steroid
Ruebhausenet al. [12]	33/F	-	2	+	+	Sterile	VAC, systemic steroid, dapsone, skin graft, amputation
Giugale et al. [13]	51/F	DM, depression, anxiety	5	-	+	Sterile	Systemic steroid
This case	42/M	RA, DM	7	+	-	Sterile	Systemic steroid

## Conclusions

In this case report, we present a case of PG that developed after carpal tunnel surgery, which is rarely reported in the literature. With this case presentation, we want to indicate the clinical and histopathological features and treatment options of postoperative PG and emphasize that PG should be considered among the differential diagnoses in rapid postoperative-onset, painful, treatment-resistant lesions. Moreover, growing with debridement should be an indicator of postoperative PG.
